# Primary Cervical Angiosarcoma Presenting With Transfusion‐Dependent Bleeding in Early Pregnancy: Diagnostic and Management Challenges

**DOI:** 10.1002/ccr3.73302

**Published:** 2026-08-07

**Authors:** Ammir Abuzahra, Ruba Atawneh, Siwar Abu Sakout, Raya Abu Ayyash, Jana Dwaik

**Affiliations:** ^1^ Department of Obstetrics and Gynecology College of Medicine, Hebron University Hebron Palestine; ^2^ Department of OB & GYNE, Faculty of Medicine Hebron University Hebron Palestine

**Keywords:** cervical angiosarcoma, cervical sarcoma, gynecologic oncology, pregnancy‐associated malignancy, transfusion‐dependent vaginal bleeding

## Abstract

Persistent vaginal bleeding during pregnancy should not be attributed solely to obstetric causes. Rare cervical malignancies, including angiosarcoma, may present with life‐threatening anemia, and MRI with early biopsy is essential to avoid diagnostic delay.

## 1 Introduction

Abnormal vaginal bleeding during pregnancy is common and most frequently attributed to benign obstetric conditions such as threatened miscarriage, cervical ectropion, or infection [1]. Consequently, malignant cervical tumors may remain unrecognized until significant morbidity develops [2]. Angiosarcoma is an aggressive malignant tumor of endothelial origin and is exceptionally rare in the uterine cervix, with only isolated cases reported in the literature [3]. Because evidence‐based management strategies are lacking, diagnosis and treatment decisions are often extrapolated from soft‐tissue sarcoma practice [4]. Early recognition is particularly important when ongoing bleeding leads to hemodynamic compromise and transfusion dependence [3]. We present a case of primary cervical angiosarcoma diagnosed during early pregnancy, highlighting the diagnostic challenges and the importance of early imaging and biopsy in patients with unexplained persistent bleeding.

## 2 Case Presentation/Examination

A 31‐year‐old multiparous woman (gravida 5, para 3, with one prior second‐trimester pregnancy loss) presented with a five‐month history of intermittent recurrent vaginal bleeding, which was occasionally heavy and associated with fatigue, dizziness, and palpitations. She also reported foul‐smelling vaginal discharge and postcoital bleeding. She denied pelvic or abdominal pain, fever, urinary symptoms, changes in bowel habits, or trauma.

Menarche occurred at 14 years of age. Her menstrual cycles were irregular, occurring approximately every 22–28 days and lasting about 8 days, with heavy menstrual flow. An intrauterine device had been in place for approximately 3.5 years but was removed because of persistent unexplained bleeding. She had not previously undergone cervical screening. The chronological sequence of the clinical events is summarized in Table 1.

**TABLE 1:** Chronological timeline of clinical events.

Time	Clinical event
5 months before admission	Recurrent intermittent vaginal bleeding
Presentation (≈12 weeks gestation)	Symptomatic anemia and evaluation
Initial assessment	Cervical mass detected on examination and ultrasound
Imaging	MRI demonstrated bulky cervical lesion; PET/CT performed after counseling for staging and excluded metastasis
Biopsy	Histopathology confirmed angiosarcoma
Multidisciplinary discussion	Decision for definitive surgical management
Surgery	Total hysterectomy and bilateral salpingo‐oophorectomy
Postoperative period	Uneventful recovery and referral for adjuvant therapy

*Note:* This table summarizes the sequence of key clinical events from initial symptom onset through diagnosis, multidisciplinary evaluation, definitive surgical management, and early postoperative course, highlighting the progression of disease and decision‐making process.

During evaluation, pregnancy was confirmed, with an estimated gestational age of approximately 12 weeks based on the last menstrual period; however, dating was uncertain because of the frequent bleeding. Her obstetric history included one second‐trimester pregnancy loss attributed to cervical insufficiency and one preterm cesarean delivery. Her medical history was otherwise unremarkable. Her surgical history included appendectomy, hemorrhoidectomy, and adenoidectomy. She reported smoking shisha for approximately one year and denied other substance use.

On examination, the patient was pale but hemodynamically stable. Abdominal examination revealed no tenderness or guarding. Speculum examination demonstrated an irregular, friable, exophytic mass arising from the cervix, with contact bleeding. The lesion occupied the external cervical os and extended into the upper vaginal canal. Bimanual examination demonstrated a bulky cervix without parametrial induration, and no adnexal masses were palpated.

The patient developed severe iron‐deficiency anemia secondary to persistent bleeding and required repeated packed red blood cell transfusions during the course of evaluation. She received 3 units when her hemoglobin level was 6.1 g/dL, increasing to 10.3 g/dL after transfusion; 2 units when her hemoglobin level was 6.2 g/dL, increasing to 8.1 g/dL; and 2 units when her hemoglobin level was 7.4 g/dL, increasing to 9.7 g/dL. Despite these transfusions, her hemoglobin level subsequently decreased again to approximately 6 g/dL, indicating persistent hemorrhage.

## 3 Methods

Persistent vaginal bleeding during pregnancy accompanied by a cervical mass raises a broad differential diagnosis including both obstetric and non‐obstetric etiologies. In the present case, several conditions were considered before histopathologic confirmation of angiosarcoma.

First, cervical carcinoma, particularly squamous cell carcinoma or adenocarcinoma, represents the most common malignant cervical tumor and frequently presents with abnormal vaginal bleeding, postcoital bleeding, or a friable cervical mass. Cervical carcinoma can also manifest during pregnancy and is therefore an important initial diagnostic consideration in patients presenting with a cervical lesion and bleeding.

Second, benign cervical lesions, including cervical polyps and cervical ectropion, may produce contact bleeding or persistent vaginal spotting during pregnancy due to increased cervical vascularity. Although these lesions are generally small and non‐infiltrative, they may mimic malignant processes clinically, particularly when bleeding is prominent.

Third, infectious cervicitis, including infections caused by *Chlamydia trachomatis* , *Neisseria gonorrhoeae* , or polymicrobial vaginal infections, may present with abnormal vaginal discharge and bleeding. However, infectious etiologies typically lack a discrete exophytic cervical mass and are usually associated with inflammatory findings rather than tumorous growth.

Fourth, benign vascular tumors of the cervix, such as cervical hemangioma, may present with vaginal bleeding and can occasionally appear as a vascular cervical mass. Distinguishing benign vascular lesions from malignant vascular tumors such as angiosarcoma can be challenging on limited biopsy specimens.

Fifth, other mesenchymal tumors of the cervix, including leiomyoma, leiomyosarcoma, and other soft‐tissue sarcomas, should be considered when a cervical mass with atypical features is identified. These tumors may demonstrate rapid growth and bleeding but lack the vasoformative architecture characteristic of angiosarcoma.

Finally, pregnancy‐related obstetric causes of bleeding, including threatened miscarriage, placental abnormalities, and subchorionic hemorrhage, are common explanations for vaginal bleeding in early pregnancy and may initially divert attention from cervical pathology.

An abdominal ultrasound performed during pregnancy documented a single viable intrauterine fetus and identified a heterogeneous cervical mass measuring approximately 5.0 × 4.5 cm; no ascites was noted. A contrast‐enhanced CT scan of the abdomen and pelvis (obtained earlier in the diagnostic workup) showed an enhancing cervical mass of roughly 3 × 3 cm, congested periuterine veins, a bicornuate uterus, mild free pelvic fluid, no pathologic lymphadenopathy, and an incidentally noted ectopic left pelvic kidney.

Subsequently, pelvic MRI with intravenous contrast demonstrated a relatively well‐defined exophytic mass arising from the external cervical os and bulging into the upper vaginal canal, measuring approximately 4.5 × 4.0 × 4.2 cm (transverse × anteroposterior × craniocaudal). The lesion showed restricted diffusion and heterogeneous avid post‐contrast enhancement. A rim of fluid signal was observed between the mass and the posterior vaginal wall, with no definite parametrial or rectal invasion. No enlarged pelvic lymph nodes were reported. The radiologic impression favored a cervical malignancy corresponding to descriptively FIGO stage IIA2 (a bulky lesion with upper vaginal involvement but without parametrial invasion).

Cervical biopsy was interpreted as a malignant vascular neoplasm. Microscopic examination demonstrated an infiltrative vasoformative neoplasm composed of irregular anastomosing vascular channels lined by atypical endothelial cells with nuclear hyperchromasia and focal multilayering. Areas of solid spindle cell proliferation and scattered mitotic figures were present without overt necrosis.

Immunohistochemical staining showed diffuse strong positivity for endothelial markers CD31 and ERG, with supportive positivity for CD34, confirming vascular differentiation. The tumor cells were negative for epithelial marker cytokeratin (AE1/AE3), as well as smooth muscle markers SMA and desmin, excluding epithelial and smooth muscle neoplasms. The proliferative index assessed by Ki‐67 was elevated, supporting malignant behavior. These morphologic and immunophenotypic findings established the diagnosis of primary cervical angiosarcoma. Nevertheless, the diagnosis of angiosarcoma was established based on characteristic morphologic features and confirmatory endothelial immunohistochemical markers (CD31, ERG, and CD34), as interpreted by experienced pathologists.

For metastatic assessment, a fluorodeoxyglucose positron emission tomography/computed tomography (FDG PET/CT) scan (from vertex to mid‐thigh) demonstrated an ill‐defined hypermetabolic cervical mass measuring approximately 3.1 × 3.7 cm with a maximum standardized uptake value (SUVmax) of 4.8, without any hypermetabolic abdominopelvic lymph nodes or distant lesions. The previously noted ectopic left pelvic kidney was again identified, but no other anomalies were seen on PET/CT.

The apparent variation in tumor size across ultrasound, CT, MRI, PET/CT, and final pathological examination most likely reflects differences in imaging modality, acquisition timing, imaging planes, and measurement technique. It is important to clarify the timing of imaging in relation to pregnancy. While initial evaluation occurred during early pregnancy, advanced imaging including FDG PET/CT was performed after multidisciplinary discussion and after the decision for definitive surgical management had been made. The patient was extensively counseled regarding potential fetal risks, including radiation exposure. Given the aggressive nature of the suspected malignancy and ongoing life‐threatening hemorrhage, prioritization of maternal health guided management decisions. Pregnancy termination was performed at the time of definitive surgery (hysterectomy), and no fetal preservation was possible.

Given the rarity of the tumor, the bulky local disease, active bleeding with transfusion‐dependent anemia, and the early gestational age, the case was discussed in a multidisciplinary tumor board including specialists in gynecologic oncology, maternal‐fetal medicine, radiation oncology, and pathology. After thorough counseling regarding the maternal oncologic risks, the morbidity from ongoing hemorrhage, and the limited pregnancy‐compatible treatment options for an aggressive pelvic malignancy, a definitive surgical approach was recommended. The patient elected to proceed with definitive surgery. She underwent an exploratory laparotomy with total hysterectomy and bilateral salpingo‐oophorectomy. At the time of surgery, the pregnancy was at approximately 12 weeks' gestation and was terminated as part of the hysterectomy procedure. Bilateral salpingo‐oophorectomy was performed as part of the definitive surgical procedure. The surgery was completed without intraoperative complications.

Gross examination revealed a hemorrhagic exophytic cervical mass measuring approximately 4.6 cm in greatest dimension. Histologic evaluation confirmed angiosarcoma involving the cervical stroma with extension to the upper vaginal mucosa. The tumor demonstrated infiltrative borders and moderate nuclear atypia consistent with intermediate‐grade malignancy. Lymphovascular invasion was identified. All surgical margins were free of tumor. The bilateral adnexa were uninvolved. These findings confirmed complete macroscopic resection with high‐risk pathologic features.

Postoperatively, an adjuvant systemic therapy regimen was recommended, consisting of a taxane‐based protocol (such as weekly paclitaxel), which has demonstrated activity in angiosarcoma. Alternative options, including anthracycline‐based regimens, were also considered based on extrapolation from soft‐tissue sarcoma treatment guidelines.

Diagnostic evaluation included serial laboratory investigations demonstrating severe iron‐deficiency anemia requiring repeated packed red blood cell transfusions. Pelvic ultrasonography identified a heterogeneous cervical mass during pregnancy. Contrast‐enhanced CT, pelvic MRI, and FDG PET/CT were subsequently performed for anatomical characterization and staging. Histopathological examination of cervical biopsy specimens, supported by immunohistochemical staining positive for CD31, ERG, and CD34 and negative for epithelial and smooth muscle markers, confirmed the diagnosis of primary cervical angiosarcoma.

Management was discussed in a multidisciplinary tumor board involving gynecologic oncology, maternal‐fetal medicine, radiation oncology, pathology, and radiology specialists. Owing to persistent life‐threatening hemorrhage, transfusion‐dependent anemia, bulky localized disease, and limited pregnancy‐compatible treatment options, definitive surgical management was recommended. The patient underwent exploratory laparotomy with total hysterectomy and bilateral salpingo‐oophorectomy, followed by referral for adjuvant taxane‐based chemotherapy and close oncologic surveillance.

In conclusion, persistent or severe vaginal bleeding during pregnancy should not be attributed solely to obstetric causes. Careful cervical examination, early imaging, and timely biopsy are essential to exclude rare malignancies such as cervical angiosarcoma, particularly when bleeding results in severe anemia or transfusion dependence. This case emphasizes the importance of multidisciplinary evaluation and individualized management when aggressive cervical malignancies present during pregnancy.

## 4 Conclusion and Results

Following definitive surgery, the patient recovered without postoperative complications. Histopathological examination confirmed primary cervical angiosarcoma with negative surgical margins, although lymphovascular invasion was present. She subsequently initiated adjuvant taxane‐based chemotherapy and, at the 2‐month follow‐up, remained clinically stable with no evidence of early recurrence. Ongoing surveillance has been planned because of the aggressive biological behavior of angiosarcoma.

At short‐term follow‐up (2 months postoperatively), the patient was clinically stable, had initiated adjuvant chemotherapy, and showed no evidence of early recurrence on initial surveillance assessment. She tolerated treatment without major complications and remains under close oncologic follow‐up.

## 5 Discussion

Primary angiosarcoma of the cervix is extraordinarily rare, and most available evidence consists of individual case reports [2]. Presenting symptoms are commonly abnormal vaginal bleeding and discharge, which overlap with far more frequent benign etiologies and with the presentation of common epithelial cervical cancers [3]. In pregnancy, such bleeding may also be attributed to threatened miscarriage, cervical ectropion, infection, or other obstetric causes, increasing the risk of diagnostic delay [4].

Fewer than 20 cases of primary cervical angiosarcoma have been reported in the literature, with even fewer occurring during pregnancy. Due to this extreme rarity, most available data are derived from isolated case reports, limiting the ability to establish standardized management strategies. Compared with previously reported cases, our patient similarly presented with abnormal vaginal bleeding but uniquely demonstrated transfusion‐dependent anemia during early pregnancy, highlighting the aggressive clinical course in this setting [4]. Unlike previously reported cases, which often presented outside pregnancy or at more advanced stages, our case uniquely highlights early gestational presentation with transfusion‐dependent bleeding, emphasizing the need for heightened clinical suspicion [1]. A brief summary of selected reported cases of primary cervical angiosarcoma is provided in Table 2 to highlight their clinical presentations, management approaches, and outcomes.

**TABLE 2:** Summary of reported cases of primary cervical angiosarcoma in the literature.

Author (year)	Age	Presentation	Tumor size	Treatment	Outcome
Shah et al. (2020) [2]	67	Postmenopausal bleeding	~4 cm	Surgery + RT	Alive, no recurrence
Betancourt et al. (2019) [3]	54	Vaginal bleeding (post‐radiation)	~3 cm	Surgery	Poor outcome
Song et al. (2024) [4]	48	Abnormal vaginal bleeding	5 cm	Surgery + chemotherapy	Alive at follow‐up
Kruse et al. (2014) [5]	Variable	Vaginal bleeding	Variable	Surgery ± chemo	Variable survival
Present case	31	Transfusion‐dependent bleeding in early pregnancy	4.5 cm	Surgery + planned chemotherapy	Stable at 2 months

*Note:* This table presents selected previously reported cases of primary cervical angiosarcoma, including patient demographics, clinical presentation, tumor characteristics, treatment modalities, and outcomes, allowing comparison with the present case.

Imaging plays an important role in local staging and treatment planning for cervical tumors. Pelvic MRI is the preferred modality for assessing tumor size, stromal invasion, parametrial involvement, and extension to the vagina or adjacent organs in cervical malignancies [3]. In this case, MRI suggested a bulky exophytic lesion with upper vaginal extension but without parametrial or rectal invasion, corresponding descriptively to FIGO stage IIA2 (used for anatomical reference only, as no standardized staging system exists for cervical angiosarcoma) [4]. PET/CT further corroborated the absence of nodal or distant disease and provided a baseline for future surveillance. Although FDG uptake was moderate, metabolic activity can be variable in low‐grade sarcomas and should be interpreted in the clinical context [1].

Although the lesion was described radiologically using the FIGO 2018 staging system for cervical carcinoma (suggestive of stage IIA2 based on upper vaginal involvement without parametrial invasion), it is important to note that angiosarcoma is a mesenchymal malignancy for which no standardized cervical‐specific staging system exists. Therefore, staging in this case was applied descriptively using conventional cervical cancer criteria to facilitate anatomical characterization, rather than to guide sarcoma‐specific management. Tumor size variation across imaging modalities (CT, MRI, and PET/CT) likely reflects differences in imaging resolution and timing. MRI was considered the most reliable modality for local staging because of its superior soft‐tissue resolution and therefore guided surgical planning [6].

Histopathologic diagnosis of angiosarcoma can be challenging, particularly in small biopsy specimens because the lesion may mimic benign vascular proliferations and other mesenchymal tumors. In the cervix, the differential diagnosis may include an atypical leiomyoma or low‐grade leiomyosarcoma, a peripheral nerve sheath tumor, and other sarcomas [7]. Thorough histological sampling and the use of endothelial immunohistochemical markers (e.g., CD31, ERG, and CD34) are typically required to confirm vascular differentiation while excluding morphologic mimics. Where available, expert gynecologic pathology consultation can be decisive for accurate diagnosis [5].

Optimal management of cervical angiosarcoma is not established due to its rarity. For angiosarcoma in general, complete surgical resection with negative margins is associated with improved local control when feasible, while systemic therapy is considered for high‐risk disease or when resection is not possible [1]. Taxane‐based chemotherapy regimens have shown activity in unresectable or metastatic angiosarcoma, and combination strategies (e.g., adding anti‐angiogenic agents) have been investigated [8]. In typical cervical carcinoma, definitive radiotherapy (with or without concurrent chemotherapy) is a mainstay of treatment; however, pelvic radiotherapy is generally contraindicated during pregnancy due to the risk of fetal radiation exposure, and chemoradiation is not feasible in early gestation [9]. In contrast, chemotherapy is typically avoided in the first trimester because of high teratogenic risk [10]. Therefore, when oncologic urgency is high, counseling may include pregnancy termination or proceeding with definitive surgery, depending on gestational age, disease stage, and patient preferences [5]. However, these principles are derived from epithelial cervical carcinoma and are not directly applicable to angiosarcoma, which follows a distinct biological behavior and is managed according to soft‐tissue sarcoma principles.

In our patient, ongoing hemorrhage with transfusion dependence, a bulky tumor with vaginal involvement, and the early stage of pregnancy effectively ruled out temporizing measures or pregnancy‐sparing treatment. Multidisciplinary counseling guided the decision to pursue definitive surgical management despite the pregnancy. Given the scarcity of cervical‐specific data, the adjuvant treatment plan was individualized to account for angiosarcoma biology and the anticipated risk of recurrence. Close surveillance is essential in such cases, because angiosarcoma is characterized by aggressive behavior and a potential for early hematogenous spread even when initial imaging shows localized disease.

This case was also notable for the incidental anatomical findings of a bicornuate uterus and an ectopic pelvic kidney, both of which may influence surgical planning and the interpretation of pelvic imaging. Awareness of urinary tract and müllerian anomalies is important for surgical safety and for correctly interpreting imaging studies in gynecologic oncology patients [11].

The main limitation of this report is the absence of detailed immunohistochemical and molecular characterization of the tumor and the lack of long‐term follow‐up at the time of writing, in addition, representative diagnostic images could not be included because the original Digital Imaging and Communications in Medicine (DICOM) files and pathology photomicrographs were no longer retrievable from the institutional archiving system at the time of manuscript revision. The original radiology reports and pathology reports were available and were used to verify all imaging and histopathological findings reported in this manuscript. No diagnostic findings were reconstructed or inferred beyond the documented medical records. All clinicopathological information presented in this report was extracted from the patient's official medical records during the original manuscript preparation.

This limitation reflects the retrospective nature of the case documentation. Nonetheless, the case adds to the very limited literature on primary cervical angiosarcoma and highlights practical considerations in staging and management, particularly when the presentation is in early pregnancy.

## Author Contributions

**Ammir Abuzahra:** conceptualization, data curation, investigation, writing – original draft, project administration. **Ruba Atawneh:** data curation, investigation, writing – review and editing. **Raya Abu Ayyash:** data curation, writing – review and editing. **Siwar Abu Sakout:** investigation, writing – review and editing. **Jana Dwaik:** writing – review and editing. All listed authors had authorized access to the clinical information necessary for preparation of this manuscript, approved the final version for submission, and accept responsibility for the accuracy, integrity, and completeness of the reported information in accordance with the ICMJE recommendations.

## Funding

The authors have nothing to report.

## Disclosure

This case report has been prepared in accordance with the CARE guidelines. All clinical information, imaging findings, operative details, histopathological diagnosis, treatment course, and follow‐up data were verified against the patient's official hospital medical records, operative notes, radiology reports, and pathology reports before manuscript submission.

The manuscript was reviewed for clinical accuracy by the treating gynecologic oncology team prior to submission. The treating gynecologic oncology team and reporting pathologists were involved in the patient's clinical care but did not participate in the study design, manuscript preparation, critical revision, or final approval of the manuscript. Consequently, they did not fulfill the ICMJE authorship criteria and were therefore not included as authors.

All listed authors had authorized access to the relevant clinical information required for preparation of this report, approved the final manuscript, and accept responsibility for the accuracy and integrity of the work.

## Consent

Written informed consent was obtained from the patient for publication of this case report. A copy of the written consent is available for review by the Editor‐in‐Chief of this journal on request. All management decisions were conducted following multidisciplinary discussion and in accordance with institutional ethical standards, with full informed consent obtained from the patient regarding oncologic treatment and pregnancy termination.

## Conflicts of Interest

The authors declare no conflicts of interest.

## Data Availability

Data available on request from the authors.
